# S100A4 as a Target of the E3-Ligase Asb2β and Its Effect on Engineered Heart Tissue

**DOI:** 10.3389/fphys.2018.01292

**Published:** 2018-09-19

**Authors:** Simon Braumann, Tilo Thottakara, Sabrina Stücker, Silke Reischmann-Düsener, Elisabeth Krämer, Julia Groß, Marc N. Hirt, Shirin Doroudgar, Lucie Carrier, Felix W. Friedrich

**Affiliations:** ^1^Cardiovascular Research Center, Institute of Experimental Pharmacology and Toxicology, University Medical Center Hamburg-Eppendorf, Hamburg, Germany; ^2^DZHK (German Centre for Cardiovascular Research), Partner Site Hamburg/Kiel/Lübeck, Hamburg, Germany; ^3^Department of Cardiology, Heart Center, Cologne Cardiovascular Research Center, University of Cologne, Cologne, Germany; ^4^Department of Cardiology, Angiology, and Pneumology, Heidelberg University Hospital, Heidelberg, Germany; ^5^DZHK (German Centre for Cardiovascular Research), Partner Site Heidelberg/Mannheim, Heidelberg, German

**Keywords:** S100A4, ubiquitin proteasome system, hypertrophic cardiomyopathy, engineered heart tissue, fibrosis

## Abstract

**Background:** S100A4 has recently emerged as an important player in cardiac disease, affecting phenotype development in animal models of myocardial infarction and pathological cardiac hypertrophy, albeit it is unclear whether S100A4 exerts a detrimental or beneficial function. The goal of the current study was to analyze S100A4 expression in models of cardiac pathology, investigate its degradation by the ubiquitin-proteasome system (UPS), and furthermore examine the functional effects of S100A4 levels in a 3D model of engineered heart tissue (EHT).

**Methods and Results:** S100A4 mRNA and protein levels were analyzed in different models of cardiac pathology via quantitative RT-PCR and Western blot, showing a higher S100A4 steady-state protein concentration in hearts of *Mybpc3*-knock-in (KI) hypertrophic cardiomyopathy (HCM) mice. COS-7 cells co-transfected with plasmids encoding mutant (MUT) Asb2β lacking the E3 ligase activity in combination with V5-tagged S100A4 plasmid presented higher S100A4-V5 protein steady-state concentrations than cells co-transfected with the Asb2β wild type (WT) plasmid. This effect was blunted by treatment with the specific proteasome inhibitor epoxomicin. Adeno-associated virus serotype 6 (AAV6)-mediated S100A4 overexpression in a 3D model of EHT did not affect contractile parameters. Immunofluorescence analysis showed a cytosolic and partly nuclear expression pattern of S100A4. Gene expression analysis in EHTs overexpressing S100A4-V5 showed markedly lower steady-state concentrations of genes involved in cardiac fibrosis and pathological cardiac hypertrophy.

**Conclusion:** We showed that S100A4 protein level is higher in cardiac tissue of *Mybpc3*-KI HCM mice probably as a result of a lower degradation by the E3 ligase Asb2β. While an overexpression of S100A4 did not alter contractile parameters in EHTs, downstream gene expression analysis points toward modulation of signaling cascades involved in fibrosis and hypertrophy.

## Introduction

S100 proteins are a group of small EF-hand calcium-binding proteins that do not have an enzymatic activity of their own but can influence a variety of fundamental molecular processes such as proliferation, migration, cell differentiation, and cardiac contractility (Donato, 2001; Zimmer et al., 2003; Donato et al., 2013). In humans the S100 protein family is comprised of 24 members, that differ from one another mostly both in the relatively small hinge region and the C-terminal extension (Donato, 2003; Zimmer et al., 2003). Upon binding of calcium S100 proteins undergo a conformational change, exposing hydrophobic pockets within the hinge region and the C-terminal extension, enabling binding of target proteins (Bresnick et al., 2015). In summary, S100 proteins act as calcium sensors that crosslink target proteins (Donato, 2001). Finally, S100 proteins are also found in the extracellular space, where they act as cell-cell-mediators in an autocrine or paracrine fashion, interacting with a variety of surface receptors, including G-protein-coupled receptors, Toll-like receptor 4 (TLR 4), receptor for advanced glycosylation end products (RAGE), and others (Donato et al., 2013). Only few members of the family have been linked to cardiac disease, among which are S100A1 and S100A4. In oncology, the importance of S100A4 is established and it is a known prognostic marker of many different malignant diseases, such as breast and colorectal cancer (Rudland et al., 2000; Liu et al., 2013). However, the role of S100A4 in the heart is less clear. Altered S100A4 expression has been described in different animal models and even humans: increased steady-state concentrations of S100A4 mRNA and protein have been found upon induction of pathological cardiac hypertrophy in rats and mice via isoprenaline, high-salt diet or transverse aortic constriction (TAC) (Inamoto et al., 2000; Tamaki et al., 2013). Moreover, in S100A4 knock-out (KO) mice the development of the hypertrophic phenotype was partially blunted, with reduced interstitial fibrosis, fewer myofibroblasts and suppressed expression of collagens and pro-fibrotic cytokines. This effect might be attributable to modulation of p53 function, indicating a primarily harmful effect of S100A4 (Tamaki et al., 2013). On the other hand, S100A4 has been suggested to have protective effects on injured myocardium since recombinant S100A4 protein has been shown to promote survival of cardiac myocytes *in vitro* after serum deprivation and treatment with doxorubicin (Schneider et al., 2007). Moreover, in a murine model of myocardial infarction S100A4 KO mice showed more adverse fibrotic remodeling and post-ischemic damage, and this effect was attenuated upon reconstitution of S100A4 protein levels after adeno-associated virus serotype 9 (AAV9)-mediated overexpression of S100A4 (Doroudgar et al., 2016). Finally, higher S100A4 protein levels were detected in human samples of hypertrophic cardiomyopathy (HCM) (Qi et al., 2016) and acute myocardial infarction (Gong et al., 2015). Intriguingly, it has yet been unclear whether S100A4 is expressed within cardiac myocytes or instead taken up in a paracrine fashion, adding to the uncertainty regarding the role of S100A4 in the heart (Schneider et al., 2007). In summary, much evidence points toward an emerging role of S100A4 in cardiac disease, albeit it is not yet clear whether S100A4 exerts a detrimental or beneficial function. Therefore, the goal of the current study was to analyze S100A4 expression in different models of cardiac pathology, investigate its degradation and gain a better understanding of its functional role after overexpression in a 3D model of engineered heart tissue (EHT).

## Materials and methods

The study complies with the Guide for the Care and Use of Laboratory Animals (NRC, 2011). Mybpc3-targeted KO and knock-in (KI) mice were developed as previously described and maintained on the Black Swiss genetic background (Carrier et al., 2004; Vignier et al., 2009). Mice with a phenotype of dilated cardiomyopathy (DCM,*LMNA*^Δ*K*32^ mice, samples kindly provided by Giséle Bonne and colleagues) were previously described (Cattin et al., 2013).

### Gene expression analysis

RNA was isolated from flash-frozen and powdered mouse and rat ventricular samples and EHTs using the Promega RNA Isolation Kit in accordance with supplier instructions. RNA concentration and purity were determined photometrically using the Nanodrop ND-1000 spectrophotometer. For gene expression in rat and mouse ventricular samples RNA (100 ng) was reverse transcribed to cDNA using the Superscript III (Invitrogen) kit. Subsequently, the cDNA was used for mRNA level quantification by real-time PCR using Power SYBR® Green PCR Master Mix and specific primers for each experiment. *Ct*-values were normalized to guanine nucleotide binding protein, alpha-stimulating (Gnas; GαS;). ΔΔ*Ct*-values were related to WT tissue.

For gene expression analysis in EHTs we used a customized NanoString's nCounter® Elements TagSet panel of 27 genes coding for proteins regulated in hypertrophy/heart failure, including Ca^2+^ and K^+^ handling proteins. Fifty nanograms of RNA of each sample were hybridized to target-specific capture and reporter probes at 67°C overnight (16 h) according to manufacturer's instructions. Samples were cooled down at 4°C, loaded into the NanoString cartridge, and the nCounter Gene Expression Assay was started immediately. Raw data were analyzed with nCounter® Sprint Profiler including background subtraction using negative controls and normalization to 6 housekeeping genes (*ABCF1, ACTB, CLTC, GAPDH, PGK1, TUBB*). Data represented the mean of normalized counts and were expressed as fold-change in EHTs overexpressing S100A4-V5 vs. control EHTs. We selected genes that were lower than 0.8-fold and higher than 1.25-fold dysregulated in EHTs overexpressing S100A4 according to Singh et al. (2017).

### Generation of plasmids

To generate plasmids for the co-transfection assay, V5-tagged S100A4 was amplified using murine fibroblasts as template. The specific primers contained *NheI* and *Not* restriction sites (5′-CACC-*NheI*-Kozak-ATG-S100A4 and 3′-S100A4-V5-*Not*) and were designed using the Primer3 online tool (http://primer3.ut.ee), for detailed primer sequences see Supplemental Table 1). After successful amplification and gel electrophoresis, the corresponding PCR fragment was gel-eluted and ligated into a pGG2-CMV-FLAG-GFP vector after removal of the FLAG-GFP insert by restriction digest. Correct ligation of the insert was assessed via colony PCR and subsequent sequencing analysis. Isolation of the plasmid was performed using the NucleoSpin® kit (Macherey Nagel). Plasmids encoding FLAG tagged Asb2β WT or MUT were used as previously described (Thottakara et al., 2015).

### Co-transfection and proteasome inhibition

Co-transfection was performed in COS-7 cells using the Turbofect™ Transfection Reagent (Thermo Fisher Scientific). According to the manual cells were seeded in 12-well plates (max. 400,000 cells/well) 24 h before transfection, reaching an 85% confluency at the time of transfection. Plasmids used were S100A4-V5 (1 μg/well) together with either Asb2β WT or MUT (500 ng/well) according to Thottakara et al. (2015). Proteasome inhibition was achieved by adding the irreversible proteasome inhibitor epoxomicin (500 nM, Calbiochem) or the vehicle dimethyl sulfoxide (DMSO, 0.05%) 48 h after transfection. Cells were harvested after 48 h and proteins were isolated for Western blot analysis.

### Western blot

Proteins were extracted from EHTs and powdered whole heart lysate of *Mybpc3*-KI mice and immunoblotting was performed as described before (Friedrich et al., 2012). Proteins were loaded on 7–10% acrylamide/bisacrylamide (37.5:1 or 29:1 BioRad) and 16.5% tris-tricine gels and then blotted onto a 0.45-μm nitrocellulose membrane (Life Technologies). Membranes were blocked in 5% milk and incubated with antibodies against S100A4 (anti-S100A4 rabbit-polyclonal, Dako A5114, 1:500), ß-actin (anti-ß-actin mouse-monoclonal, 1:20,000), GAPDH (anti-GAPDH mouse-monoclonal, HyTest 5G4-6C5, 1:5,000), FLAG (anti-FLAG mouse-monoclonal, Sigma F3165, 1:5,000), V5 (anti-V5 mouse-polyclonal, Invitrogen PA1-993, 1:1,000), GFP (anti-GFP rabbit-polyclonal, Abcam ab6556, 1:2,000), and total ERK (anti-Total ERK rabbit-polyclonal, Cell Signaling #9102, 1:2,000). Secondary antibodies were coupled to HRP (Sigma). Signals were detected by ECL Plus Western blotting detection system substrate (Amersham GE Healthcare Life Sciences, Munich, Germany). Quantification of the signal was determined using Gene Tools software (Syngene, Cambridge, UK). S100A4-protein levels were normalized to ß-actin, GAPDH, or total ERK, as mentioned above.

### Generation of AAV6 (S100A4, GFP, empty vector)

To generate adeno-associated virus serotype 6 (AAV6), generation of a V5-tagged S100A4 was performed as described above (see section Generation of Plasmids). To ensure cardiac myocyte specific expression in the EHT, the insert was then ligated into the pGG2-cTnT-GFP vector after removing the GFP, which was framed by restriction sites for *NheI* and *Not*. This vector contained the human cardiac troponin T promoter (cTnT, Supplemental Figure 1) and had also been used previously (Gedicke-Hornung et al., 2013; Mearini et al., 2013, 2014; Prondzynski et al., 2017). The empty vector (EV) was designed by removing the GFP-insert and re-ligating the vector backbone. All cloning steps were verified by sequencing. The AAV6 production was performed by the HEXT Vector Core Unit, Department of Experimental Pharmacology and Toxicology, University Medical Center Hamburg-Eppendorf, 20246 Hamburg, Germany. AAV6 titers ranged from 9.77 × 10^11^ to 1.70 × 10^12^ virus genomes per ml (vg/ml).

### Generation and transduction of EHT with AAV6

EHTs were generated from neonatal rat cardiac cells as previously described using a 24-well format (Hansen et al., 2010; Hirt et al., 2012; Crocini et al., 2013). Briefly, ventricular heart cells (the atria were carefully excised) from neonatal Wistar rats (postnatal day 0–3) were isolated by a fractionated DNase/Trypsin digestion protocol. This procedure was reviewed and approved by the Ethics Commission of the Medical Association of Hamburg. Rat ventricular heart cells, fibrinogen, thrombin, and DMEM (2 ×, to match the volumes of fibrinogen and thrombin and thus ensuring isotonic conditions) were mixed and pipetted into molds, which were obtained by casting 2% agarose (in PBS) around Teflon® (polytetrafluoroethylene) spacers in a 24-well culture dish. Afterload enhancement was performed by inserting a small metal brace in between two silicone posts, thus increasing resistance between two adjacent silicone posts by factor 12. Phenylephrine was added in a concentration of 20 μml/l. Both interventions started 14 days after EHT generation and lasted for a total of 7 days as described in detail by Hirt et al. (2012).

EHTs were transduced with AAV6 encoding either S100A4-V5 or EV by adding the AAV6 to the initial master mix according to its titer before the master mix was pipetted into the molds. The MOI of 1,000 was chosen based upon previous feasibility and viability experiments using an AAV6-GFP as shown in Crocini et al. (2013). GFP transduction of EHTs was successfully repeated as a proof of concept (data not shown). After polymerization of fibrin (1–2 h), EHTs were transferred to new cell culture dishes filled with medium. Spontaneous beating of EHTs started 7 days after casting. Contraction measurements were performed on day 10, 13, 15, 17, 20, and 22, as previously described (Hansen et al., 2010; Schaaf et al., 2011). Briefly, a customized software analyzed 30 s videos of spontaneously beating EHTs calculating force, contraction and relaxation velocities (CV and RV) and time from 20% of contraction to peak contraction (time to peak, TTP −80%) and time from peak contraction to 20% of contraction, i.e., relaxation time (RT 80%) (**Figure 4D**). After 22 days, EHTs were PBS washed three times and directly processed or frozen in liquid nitrogen.

### Immunofluorescence

For immunofluorescence analysis, whole mount EHTs were used. EHTs were fixed overnight at 4°C using Histofix® (Carl Roth) solution and washed for 15 min using Tris-buffered saline (TBS). EHTs were then removed from the silicone posts and treated with a blocking solution (TBS 0.05 M, pH 7.4, 10% FCS, 1% BSA, 0.5% Triton X-100) for 24 h at 4°C. Then, EHTs were incubated with primary antibodies against V5 (Invitrogen) and titin M8/M9 (Novus Biologicals). Secondary antibody staining was done with Alexa-fluor 488 anti-mouse and Alexa-fluor 546 anti-rabbit (Invitrogen, Life Technology, Darmstadt, Germany, 1:600). Nuclei were stained with DRAQ5TM (DR50050, Biostatus Limited®, 1:1,000). For examination, non-fluorescing Fluoromount G medium was added and EHTs were mounted on indentated microscopic slides. Signals were visualized with a Carl Zeiss confocal microscope (Zeiss LSM 510 META). Confocal images were recorded using a Zeiss LSM 5 Image System.

### Statistical analysis

Data were expressed as mean ± SEM. Statistical differences were analyzed using the one-way or two-way analysis of variance (ANOVA) followed by the Bonferroni adjustment for *post-hoc* multiple comparison, or by the unpaired Student's *t*-test, as indicated in the legend of each figure. A *p*-value of <0.05 was considered to be statistically significant.

## Results

### S100A4 mRNA is differentially regulated in mouse models of DCM and HCM and engineered heart tissue hypertrophy models

**Figure 1:**
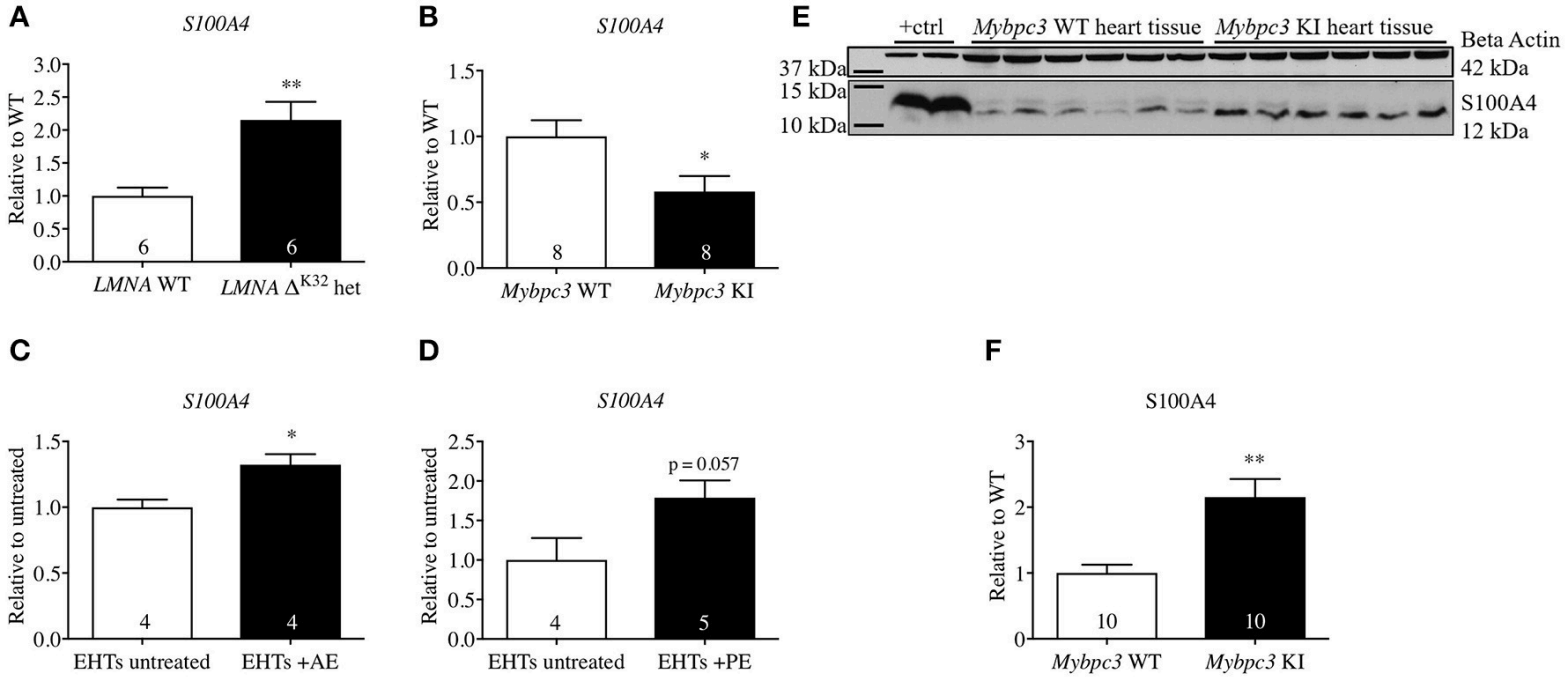
Steady-state concentrations of S100A4-mRNA or protein in different models of cardiac pathology. **(A)** Dilated cardiomyopathy in *LMNA-*Δ*K32*-heterozygous (het) vs. wild type (WT) mice at 1 year of age, **(B)** hypertrophic cardiomyopathy in *Mybpc3*-KI vs. WT mice at 10 weeks of age, **(C)** pathological cardiac hypertrophy in EHTs with afterload enhancement (AE, **C)** according to Hirt et al. (2012) **(D)**. **(E)** Representative Western blot and **(F)** quantification of S100A4 protein steady state concentrations in *Mybpc3*-KI and WT heart tissue. Neonatal rat ventricular myocyte whole cell lysate served as positive control in **(E)** lanes 1 + 2. Data are expressed as mean ± SEM. ^*^p < 0.05, ^**^p < 0.01, vs. WT mice, untreated EHTs, or WT murine heart tissue, unpaired Student's *t*-test, sample numbers are indicated in the bars.

Different and partly conflicting functional roles of S100A4 in cardiac tissue have been described. *S100A4* mRNA level was elevated in different animal models of cardiac diseases such as acute afterload enhancement and acute myocardial infarction in rats (Schneider et al., 2007) and mice (Schneider et al., 2007; Tamaki et al., 2013; Doroudgar et al., 2016). To broaden the data on S100A4 expression in cardiac disease models we analyzed steady-state concentrations of *S100A4* mRNA in animal models mimicking HCM (*Mybpc3*-targeted KI mice) or dilated cardiomyopathy (DCM, *LMNA*^Δ*K*32^ mice) as well as an *in vitro* hypertrophy 3D model of EHTs (treated with afterload enhancement, AE, or phenylephrine, PE) according to Hirt et al. (2012). *LMNA*^Δ*K*32^ mice carry a lysine 32 deletion in *LMNA (*LMNA^Δ*K*32^), encoding lamin A/C. At 1 year of age heterozygous LMNA^Δ*K*32^-mice display lower heart weight and at the same time increased end-systolic and end-diastolic diameters, presenting a DCM-like phenotype (Cattin et al., 2013). The *Mybpc3*-targeted KI HCM mice carry a point mutation, which is associated with a severe phenotype and a poor prognosis in humans (Richard et al., 2003). Homozygous KI mice develop cardiac dysfunction and hypertrophy shortly after birth (Vignier et al., 2009; Fraysse et al., 2012; Mearini et al., 2014) and exhibit increased proteasome activities in early postnatal development and impairment of the ubiquitin-proteasome system (UPS) in 1-year-old mice (Schlossarek et al., 2012). Corresponding to previous findings of increased *S100A4* mRNA in diseased heart tissue, we found significantly higher levels of *S100A4* mRNA in hearts of 1-year-old LMNA^Δ*K*32^-mice compared to corresponding wild type (WT) controls (Figure 1A). In the *Mybpc3*-KI animals, the steady-state S100A4 mRNA concentration was significantly lower (Figure 1B), whereas steady-state S100A4 protein concentration was 2.6-fold higher than in WT controls (Figures 1E,F). Similarly, S100A4 steady-state mRNA concentration was higher in 3D EHT model submitted to afterload enhancement (Figure 1C), whereas it was non-significantly higher in the EHTs treated with phenylephrine compared to untreated controls (*p* = 0.57; Figure 1D).

### Asb2β targets S100A4 for proteasomal degradation

**Figure 2:**
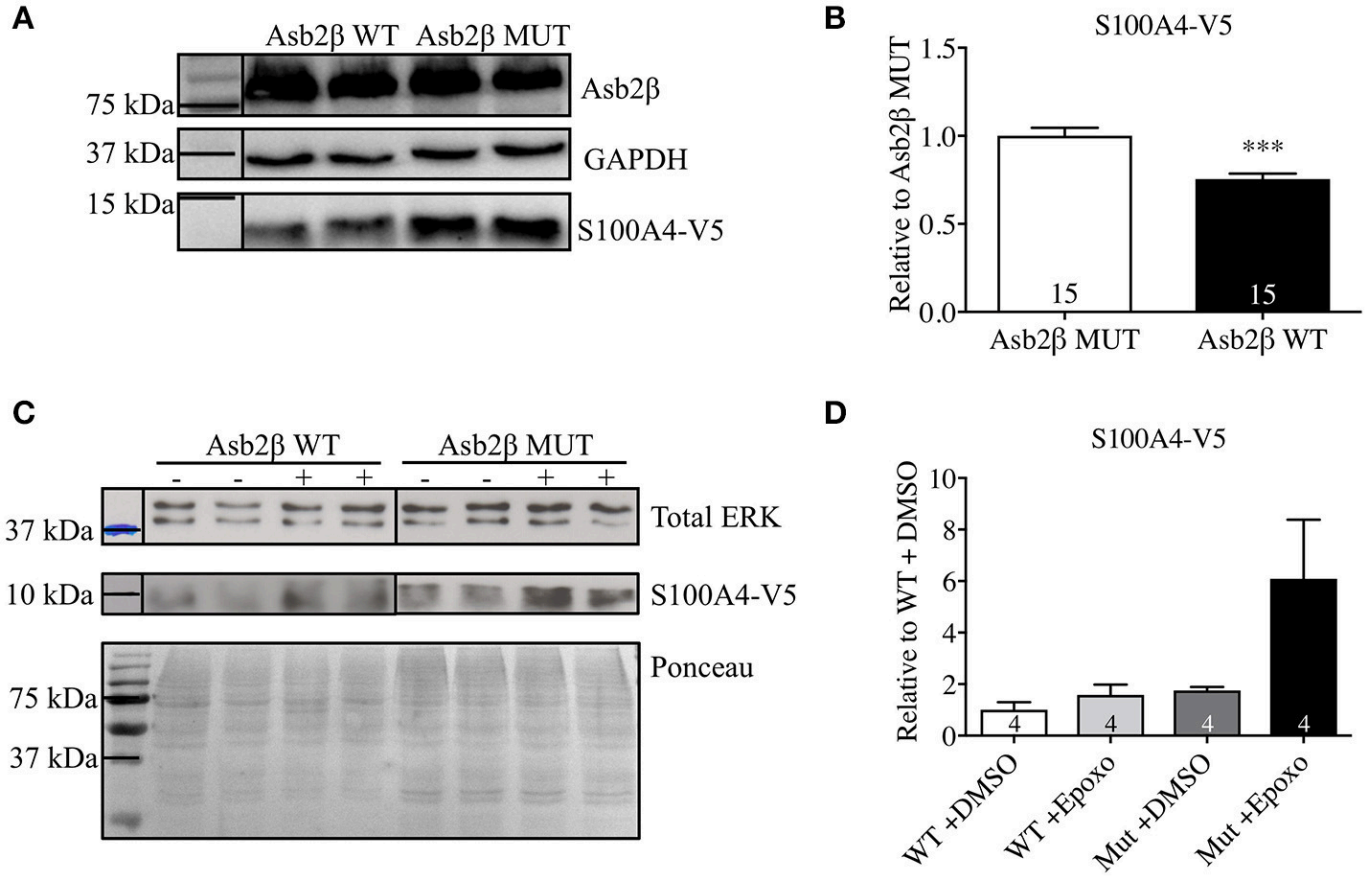
S100A4 protein levels in COS-7-cells. **(A)** Representative Western Blot and quantification **(B)** of S100A4-V5 protein level in COS-7-cells co-transfected with either Asb2β-WT or -MUT isoform. **(C)** Representative Western blot normalized to total ERK expression. Ponceau staining is shown for demonstration purposes. **(D)** Quantification of S100A4-V5 protein level in COS-7-cells co-transfected with Asb2β-MUT treated either with epoxomicin a specific and irreversible inhibitor of the proteasome indicated as (+) or vehicle control (–). Data are expressed as mean ± SEM. ^***^p < 0.001, vs. Asb2β WT or vehicle control treated COS-7-cells, unpaired Student's *t*-test, sample numbers are indicated in the bars.

Our group has previously shown that the UPS is impaired in *Mybpc3-*KI mice (Schlossarek et al., 2012) and that among many other ubiquitin E3 ligases, Asb2β is downregulated the most in these mice (Thottakara et al., 2015). We previously demonstrated that consequently protein steady-state concentrations of desmin, a target of Asb2β, were higher in *Mybpc3-*KI mice. Since S100A4 mRNA steady-state concentration was lower, but protein steady-state concentration was higher in *Mybpc3*-KI mouse hearts, we examined whether Asb2β also targets S100A4 for proteasomal degradation. COS-7 cells were co-transfected with plasmids expressing a V5-tagged S100A4 (S100A4-V5) and Flag-tagged Asb2β-WT or a mutant form (MUT) lacking ligase activity. S100A4-V5 protein levels were significantly lower when S100A4-V5 was co-expressed together with Asb2β-WT compared to co-expression with Asb2β-MUT (Figures 2A,B). Incubation with the proteasome inhibitor epoxomicin markedly attenuated this effect and stabilized S100A4-V5 expression in the Asb2β-WT group (Figures 2C,D).

### AAV6-S100A4 transduction leads to specific protein overexpression of S100A4

**Figure 3:**
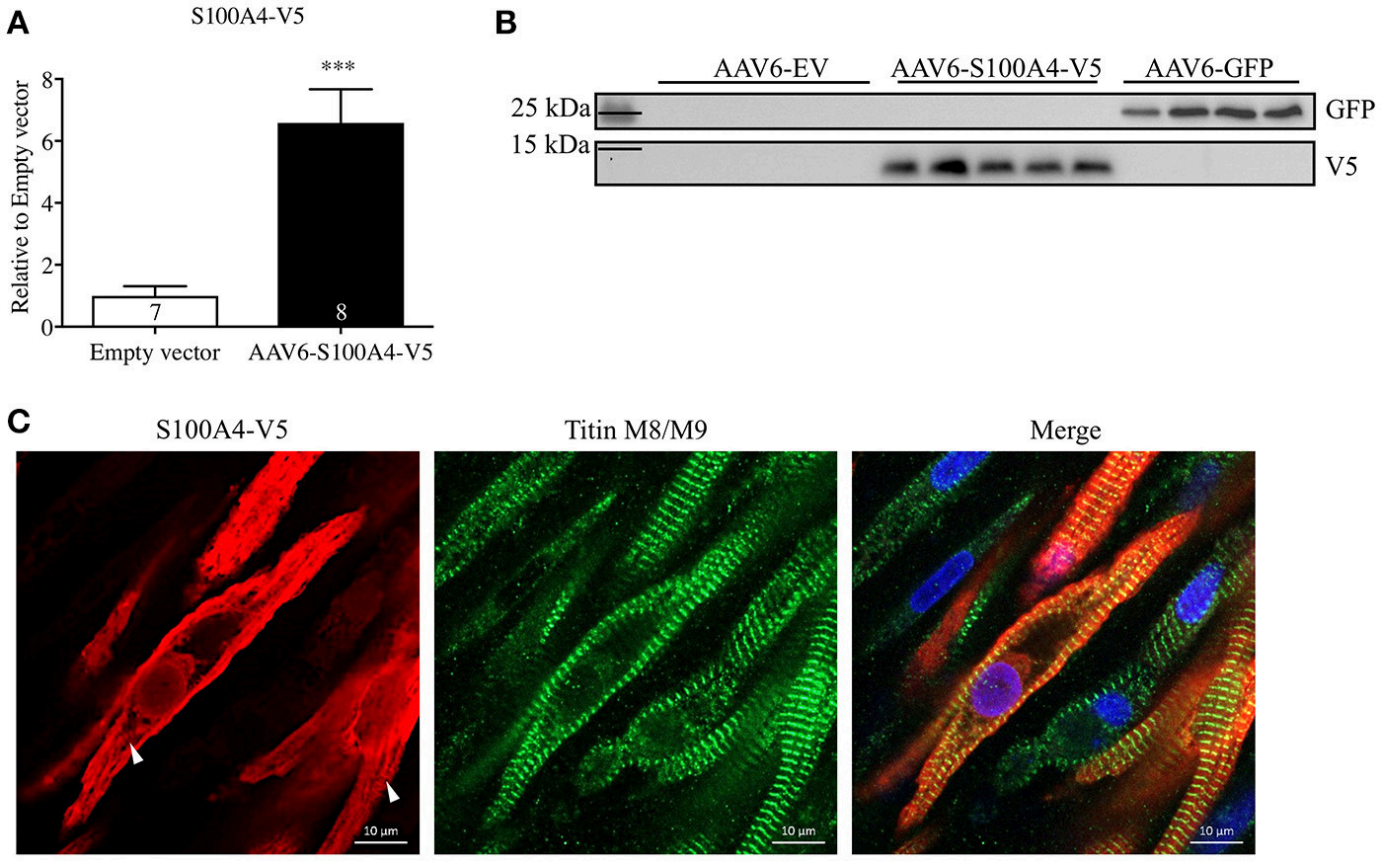
S100A4 mRNA and protein levels after AAV6-mediated overexpression of S100A4-V5 in rat EHT. **(A)** After 22 days EHTs were harvested and processed accordingly, S100A4 levels were determined by RT-qPCR and related to EV. **(B)** Western blot analysis with antibodies directed against the V5 tag and GFP. GFP transduced EHTs were used as positive control. To exclude effects associated with AAV6-treatment, a control group was treated with an empty vector (EV). Molecular weight markers (MW) are indicated. **(C)** Immunofluorescence: After fixation EHTs were stained with antibodies against the V5-epitope (red) or titin M8/M9 (green). Nuclei were stained with DRAQ5 (blue). White arrows indicate partially striated S100A4-V5 signal. ^***^p < 0.001 vs. EV, unpaired Student's *t*-test, sample numbers are indicated in the bars. Scale bars 10 μm (63x magnification).

To examine the effect of S100A4 on cardiac contractile function, we overexpressed S100A4 (MOI 1000) in our 3D model of EHT to assess if S100A4 has an influence on cardiac function without the prerequisite of a pathologic phenotype. For EHT transduction we used an AAV6 expressing the S100A4-V5 under the control of human cardiac troponin T (*TNNT2*). In order to prove a successful expression of the tagged construct, we evaluated mRNA and protein levels of S100A4 via quantitative real time PCR and Western blot, respectively. To exclude side effects associated with AAV6-treatment itself, a control group was transduced with an EV carrying no cDNA. Successful transduction of EHTs was shown by a 6.6-fold higher *S100A4* mRNA steady-state concentration (Figure 3A) and a marked increase in protein amount (Figure 3B). It is important to note, that the primers used for qPCR (Figure 3A) detect both endogenous S100A4 and the transduced, exogenous construct whereas the V5-antibody was directed specifically against the exogenous construct. GFP expression served as positive control in protein analysis (Supplemental Figure 3). Immunofluorescence analysis of transduced EHT showed typical striations with titin M8/M9 antibody, but no co-localization with the S100A4-V5 signal was seen (Figure 3C). The signal for S100A4-V5 was mainly cytoplasmic, sometimes indicating a striated pattern, whereas the nuclear signal was less intense. Parts of the cytoplasm which looked like vesicle-like structures were negative. Also, the conical myofibril-free myoplasm region around the nucleus showed no V5 signal.

### S100A4 overexpression does not affect the EHT contractile function

**Figure 4:**
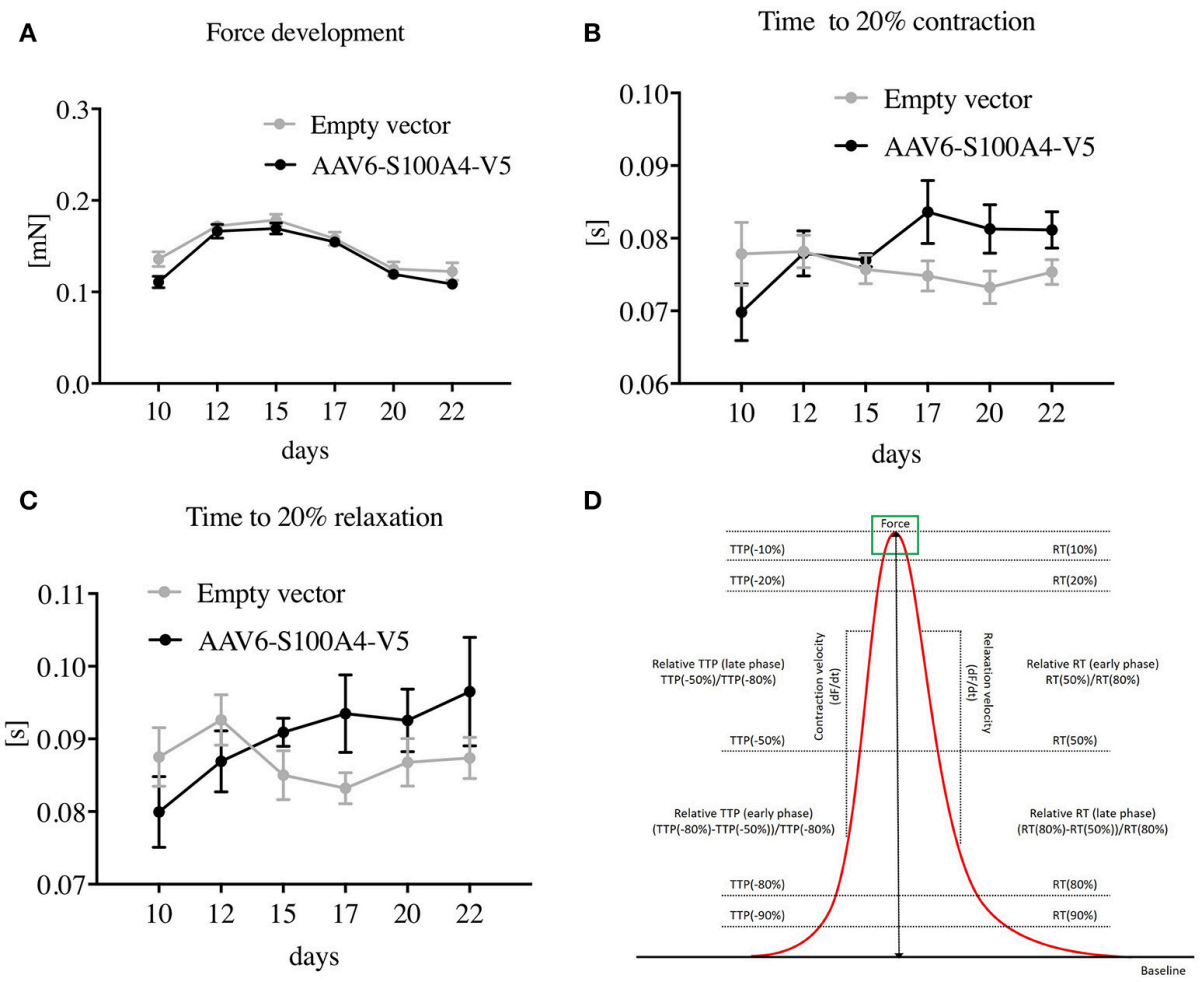
Functional effects of AAV6-mediated overexpression of S100A4 in EHT. **(A)** Force development, **(B)** time from 20% of contraction to peak contraction (time to peak, TTP −80%), and **(C)** time from peak contraction to 20% of contraction, i.e., relaxation time (RT 80%) all compared to EHTs treated with an empty vector to exclude side effects of the treatment. Illustration of the functional parameters **(D)**. Data are expressed as mean ± SEM. *One-way*-*ANOVA* with *Dunnett's-Multiple-Comparison*-*Test, n* = 43–57 per group.

We then determined functional parameters of S100A4-V5 transduced EHTs such as force, contraction, and relaxation velocities (CV and RV) and time from 20% of contraction to peak contraction (time to peak, TTP −80%) and time from peak contraction to 20% of contraction, i.e., relaxation time (RT 80%), using an optical measurement system (Hansen et al., 2010; Friedrich et al., 2012, 2014). The functional parameters were assessed 10–22 days after EHT formation. Analysis of contractile function revealed that S100A4 overexpression did not alter EHT contractile performance. Neither force development (Figure 4A), CV, RV (not shown) nor TTP −80% or RT 80% (Figures 4B,C) differed between EHTs transduced with S100A4-V5 and EHTs transduced with the EV.

### S100A4 overexpression leads to decreased mRNA concentrations of pro-hypertrophic and pro-fibrotic genes

**Figure 5:**
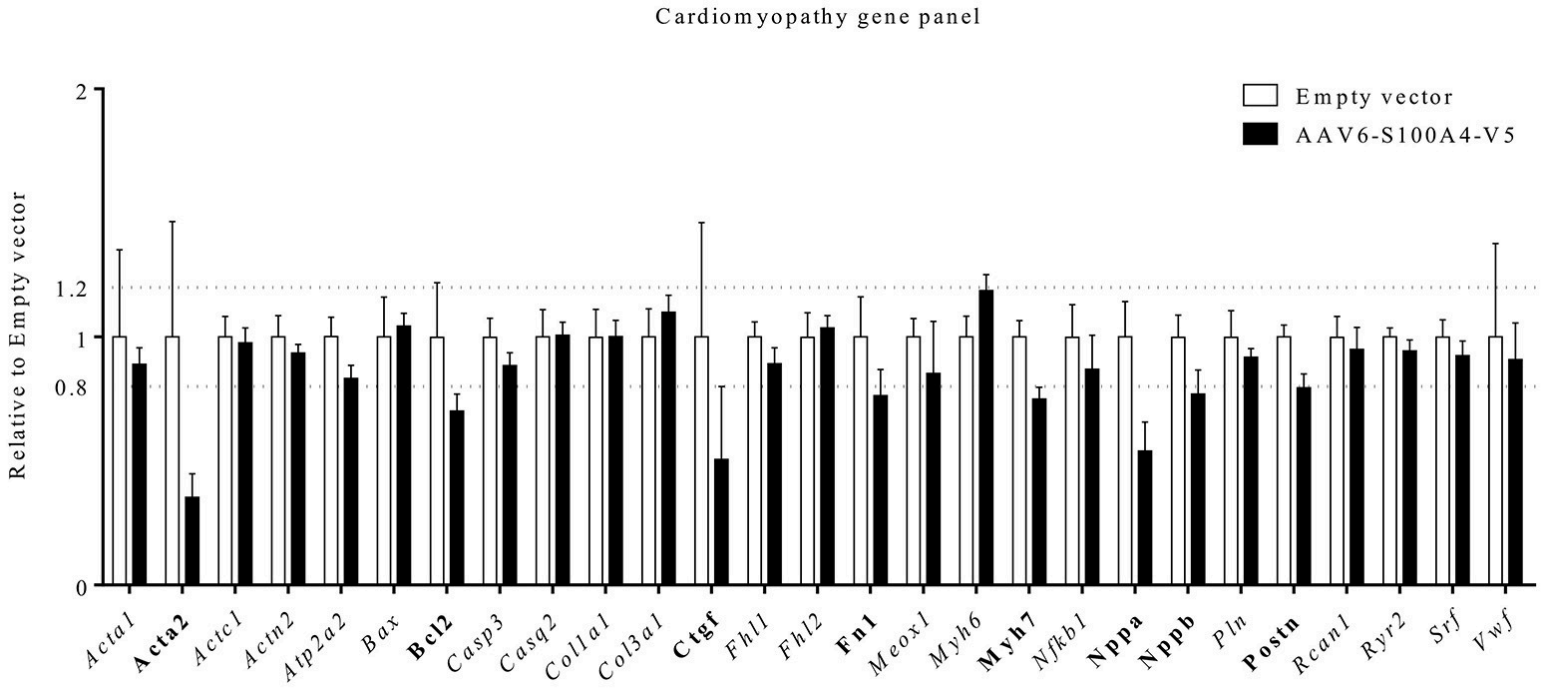
Gene expression analysis. Using a customized NanoString's nCounter® Elements TagSet panel of 27 genes coding for proteins regulated in hypertrophy/heart failure, including Ca^2+^ and K^+^ handling proteins, steady state mRNA-concentrations were analyzed in 22 days old EHTs after AAV6-mediated overexpression of S100A4 (*n* = 6) under the control of the human cardiac troponin T promoter (*TNNT2)* or EHTs after treatment with an empty vector (*n* = 5). Data were analyzed with nCounter® Sprint Profiler including background subtraction using negative controls and normalization to 6 housekeeping genes (*ABCF1, ACTB, CLTC, GAPDH, PGK1, TUBB*). Data represented the mean of normalized counts and were expressed as fold-change in EHTs overexpressing S100A4V5 vs. control EHTs. A threshold of −0.2- and +0.25-fold difference related to empty vector was considered a relevant result and such genes were marked in bold.

S100A4 KO mice showed an ameliorated phenotype and a blunted upregulation of fibrosis-associated genes *Col1a1, Col3a1, Fn1*, and *Ctgf* [encoding type I collagen, type III collagen, Fibronectin, and connective tissue growth factor (CTGF)] after TAC (Tamaki et al., 2013). To investigate whether S100A4 overexpression has an influence on expression of genes associated with fibrosis and hypertrophy, we analyzed steady-state concentrations of a custom-made gene panel of genes dysregulated in pathological hypertrophy and fibrosis. A threshold of −0.2 and +0.25-fold difference relative to EV was considered a relevant result according to Singh et al. (2017). S100A4 overexpression in EHT led to a markedly lower steady-state concentration of genes associated with pathological hypertrophy in EHTs, such as *Myh7, Nppa, Nppb*, encoding β-myosin-heavy-chain (β-MHC), natriuretic peptide B (BNP), and natriuretic peptide A (ANP). Compared to EV EHTs S100A4-transduced EHTs also presented lower steady-state concentration of profibrotic genes *Acta2, Fn, Ctgf*, and *Postn*, encoding α-smooth muscle actin (α-SMA), fibronectin, CTGF, and periostin (Figure 5).

## Discussion

In the present study we examined S100A4 steady-state concentration in different experimental models of cardiac pathology, investigated its degradation by the UPS and analyzed its functional effect following overexpression in EHTs, including potential downstream effects on gene expression level of cardiomyopathy-associated genes. Our main findings were as follows: (i) *S100A4* steady-state mRNA concentration was decreased in *Mybpc3*-KI HCM mice, but increased in *LMNA*^Δ*K*32^ DCM mice and two 3D EHT models of experimental pathological hypertrophy; (ii) S100A4 steady-state protein concentration was higher in *Mybpc3*-KI HCM mice; (iii) The E3 ubiquitin ligase Asb2β targeted S100A4 for proteasomal-mediated degradation *in vitro* in a COS-7 cell assay; (iv) Stable AAV6-mediated overexpression of S100A4 in rat EHTs had no effect on contractile function, but led to lower mRNA steady-state concentrations of genes associated with hypertrophy and fibrosis.

Increased steady-state concentration of *S100A4* mRNA and protein levels have been found in pathologically hypertrophied and failing hearts of rats induced by aortic banding, myocardial infarction (Schneider et al., 2007) as well as TAC, or appropriate diet in Dahl salt-sensitive animals (Tamaki et al., 2013). Upon myocardial infarction induced by left anterior descending (LAD) coronary artery ligation, mice expressed higher amounts of S100A4 (Doroudgar et al., 2016). As explained earlier, the interpretation of these results differs from one another. Intriguingly, S100A4 transcript levels did not increase in every model of cardiac pathology. Whereas increased steady-state concentrations of *S100A4* mRNA were found in a DCM mouse model and two 3D EHT models of experimental pathological hypertrophy, it was significantly lower in *Mybpc3*-KI HCM than in control mouse hearts. However, when we analyzed S100A4 cardiac protein levels we found a nearly 3-fold increase compared to WT controls indicating that the reduced steady-state concentration of *S100A4* mRNA could be a response to higher protein levels. It should be noted that the age of the examined murine models differs from one another. Considering changing UPS activity over increasing age (Schlossarek et al., 2012) this has to be remembered when interpreting the different mRNA steady-state concentrations of *S100A4*.

The UPS is a canonical protein quality control system and its dysregulation has been associated with the development of HCM in mice and probably also in humans (Predmore et al., 2010; Schlossarek et al., 2012). We have previously shown that Asb2β is the most downregulated E3 ubiquitin ligase in HCM *Mybpc3*-KI mice and that it targets desmin for proteasomal degradation (Thottakara et al., 2015). Interestingly, S100A4 also seems to be a target of Asb2β. Together these results indicate that the lower UPS-mediated S100A4 degradation due to lower Asb2β expression could be the reason for the higher S100A4 protein steady-state concentrations in our *Mybpc3*-KI mice.

*Mybpc3*-KI mice exhibit systolic and diastolic dysfunction (Fraysse et al., 2012). As mentioned before, it is still unclear if increased S100A4 expression is the underlying cause of or rather a protective reaction upon the development of a pathological phenotype. We therefore overexpressed S100A4 in rat cardiac cell EHTs. This did not alter functional development of the EHT, pointing against a primarily harmful effect of S100A4 on contractile function at least in our 3D EHT model which has no basal phenotype. We furthermore tried two other MOI (100 and 300), which also did not affect contractile EHT function (Supplemental Figure 2). Therefore, we posit that the contractile cardiac dysfunction of the *Mybpc3*-KI mice is not directly attributable to higher S100A4 protein expression. To further analyze potential downstream effects after S100A4 overexpression, we first examined the localization of S100A4-V5. S100A4 has been reported to be expressed in many different interstitial cells such as fibroblast-like cells, macrophages, endothelial cells, and smooth muscle cells (Schneider et al., 2007). It is also highly expressed in embryonic and postnatal cardiac myocytes, but has been reported to be absent in healthy adult cardiac myocytes (Doroudgar et al., 2016). Interestingly, this changes dramatically upon myocardial injury or hypertrophy when S100A4 expression increases (Schneider et al., 2007; Tamaki et al., 2013; Doroudgar et al., 2016). After overexpression of S100A4 in EHTs, the protein was found mainly in the cytosol and with a lesser intensity in the nucleus of cardiac myocytes. These findings correspond to previous publications where it was also found within the cytoplasm and nucleus (Wen et al., 2013; Doroudgar et al., 2016). Interestingly, a conical region around the nucleus, presumably the perinuclear endoplasmic reticulum (ER), did not show any S100A4 expression. This is consistent with the fact that S100A4 does not contain an ER-targeting or ER retention sequences, and therefore is not an ER-targeted protein. In the cytosol, S100A4 interacts with myofibrils like actin to reorganize the cytoskeleton (Helfman et al., 2005). The region around the nucleus is free of myofibrils, providing a further explanation for the lack of S100A4 localization in this area. Noteworthy is the fact that no co-localization with the M-band region of titin, but in some cells an alternating striated pattern, indicating a Z-band localization, was seen. As Asb2β is known to be localized in the Z-band of the sarcomere (Thottakara et al., 2015) and we propose S100A4 to be a target of Asb2β, this further supports our hypothesis that S100A4 is a target of Asb2β.

Myocardial fibrosis is another important feature of HCM, known to be of prognostic relevance in humans (He et al., 2017). Myofibroblasts are central actors in the development of fibrosis. They arise from heterogenous origins upon tissue injury, including resident fibroblasts, epithelial mesenchymal transition (EMT) but also endothelial mesenchymal transition (ENT) (Hinz et al., 2007; Zeisberg et al., 2007; Davis and Molkentin, 2014). This transdifferentiation is at least in part mediated by fibronectin and periostin (Tomasek et al., 2002; Crawford et al., 2015), while CTGF is able to maintain a profibrotic environment for the activated myofibroblast (Leask, 2010). A key feature of terminally differentiated myofibroblasts is the expression of α-SMA (Hinz et al., 2007). S100A4 is believed to be a common regulator of connective tissue regeneration in many different organs, including EMT and ENT. S100A4 is more abundantly expressed in fibroblasts than in cardiomyocytes (Doroudgar et al., 2016). Intriguingly, while this seems to contribute to disease progression in non-cardiac connective tissue pathologies in lungs and kidney (Greenway et al., 2004; Bruneval et al., 2005), the effect of S100A4 in the heart seems to be cardioprotective (Schneider et al., 2007). In EHTs overexpressing S100A4 we found markedly reduced steady-state mRNA-concentrations of *Acta2, Ctgf*, *Fn1*, and *Postn*, (Figure 5). Together these results indicate that overexpression of S100A4 could have supressed myofibroblast activity in our EHTs.

Another defining feature of HCM is cardiac hypertrophy. S100A4 is also involved in this process as it promotes cardiac hypertrophy *in vitro* (Schneider et al., 2007). Reactivation of the fetal gene program is a hallmark feature of cardiomyocytes in response to a variety of pathological conditions including pathological cardiac hypertrophy (Taegtmeyer et al., 2010). Classical fetal genes among different species include *Nppa* and *Nppb* (Taegtmeyer et al., 2010). The myosin heavy chain (MHC) is part of the contractile apparatus in the sarcomere and consists of two isoforms, α-MHC, encoded by *Myh6* and β-MHC, encoded by *Myh7*. In failing rodent hearts a switch in isoform expression toward *Myh7* is seen and this switch is a common feature of the fetal gene program as well (Feldman et al., 1993). Our EHTs overexpressing S100A4 showed lower levels of *Nppa, Nppb*, and *Myh7*, while *Myh6* expression tended to be higher. Together these results show that S100A4 overexpression led to a decreased activation of the fetal gene programm in our EHTs. Both findings, i.e., reduced steady state mRNA levels of mediators of fibrosis and hypertrophy are in line with recently reported data from Doroudgar and colleagues, who have shown more adverse fibrotic remodeling, more post-ischemic damage and increased *Nppa* and *Nppb* transcription in S100A4 KO mice (Doroudgar et al., 2016) and from Qian and colleagues, who have shown that siRNA-mediated downregulation of S100A4 alleviates cardiac fibrosis after LAD-ligation in mice (Qian et al., 2018).

This study has several limitations. The degradation assay and S100A4 overexpression in EHT were both *in vitro* experiments. Our standardized 3D-EHT-model has been extensively evaluated (Hansen et al., 2010; Schaaf et al., 2011; Hirt et al., 2012), yet it obviously does not resemble a complete heart. Further studies analyzing S100A4 protein levels after AAV-mediated overexpression of Asb2β in healthy and *Mybpc3* KI mice but also analyzing *Mybpc3* KI phenotype after overexpression of S100A4 are wanted. The high S100A4 expression in COS-7-cells co-transfected with Asb2β-MUT and treated with epoxomicin could indicate that another E3-ligase is critically involved in S100A4 degradation or that Asb2β might target a protein upstream of S100A4 critical for its expression. Lastly, S100A4 overexpression was performed in EHTs without a baseline pathologic phenotype.

In summary, we have shown that S100A4 protein steady-state concentration is higher in *Mybpc3*-KI mice, likely due, at least in part, to a reduced activity of the UPS. While an overexpression of S100A4 did not alter contractile parameters in EHTs, downstream analysis points toward a beneficial effect on expression of genes associated with fibrosis and hypertrophy.

## Author contributions

SB: execution of experiments, analysis, and interpretation of data, figure preparation, writing, and drafting the manuscript. TT: preliminary data analysis. SS: isolation and treatment of cardiac muscle strips, execution of experiments. SR-D, EK, and JG: execution of experiments. MH, SD, and LC: analysis and interpretation of data, correction of the manuscript. FF: conception and design of research, execution of experiments, analysis, and interpretation of data, figure preparation, drafting of the manuscript. All authors critically discussed the results and reviewed and approved the manuscript before submission.

### Conflict of interest statement

The authors declare that the research was conducted in the absence of any commercial or financial relationships that could be construed as a potential conflict of interest.

## Supplementary material

Supplemental Figure 1 Map of the plasmid used for the generation of AAV6, containing the C-terminally V5-tagged construct under the control of the human cardiac troponin T promoter (*TNNT2)*. SV40_polyA sequence leads to polyadenylation to prevent rapid degradation, the two AAV_ITRflop sequences (*inverted terminal repeats*) are essential for AAV synthesis, the f1_origin allows for optional siRNA construction.

Supplemental Figure 2 Functional effects of AAV6-mediated overexpression of S100A4 (MOI 100, **A–C** and MOI 300, **D–F**) under the control of the human cardiac troponin T promoter (*TNNT2)* in engineered heart tissue (EHT) compared to EHTs treated with an empty vector to exclude side effects of the treatment. **(A,D)** Force development, **(B,E)** time to 20% contraction, i.e., TTP (−80%) or **(C,F)** relaxation, i.e., RT (80%). Data are expressed as mean ± SEM. *One-way*-*ANOVA* with *Dunnett's-Multiple-Comparison*-*Test, n* = 4–9 per group.

Supplemental Figure 3 Uncropped Western blots of Figure 1E. On the left staining for S100A4 (anti-S100A4 rabbit-polyclonal, Dako A5114, 1:500) on the right staining for ß-actin (anti-ß-actin mouse-monoclonal, 1:20,000).

Supplemental Table 1 Sequences of primers chosen for amplification of V5-tagged S100A4 insert, later used for construction of respective plasmid and AAV6.
