# Supplementary figures and images for: S100A4 as a Target of the E3-Ligase Asb2β and Its Effect on Engineered Heart Tissue
Source: Front Physiol. 2018 Sep 19;9:1292. doi: 10.3389/fphys.2018.01292 (PMC6157440; doi:10.3389/fphys.2018.01292)

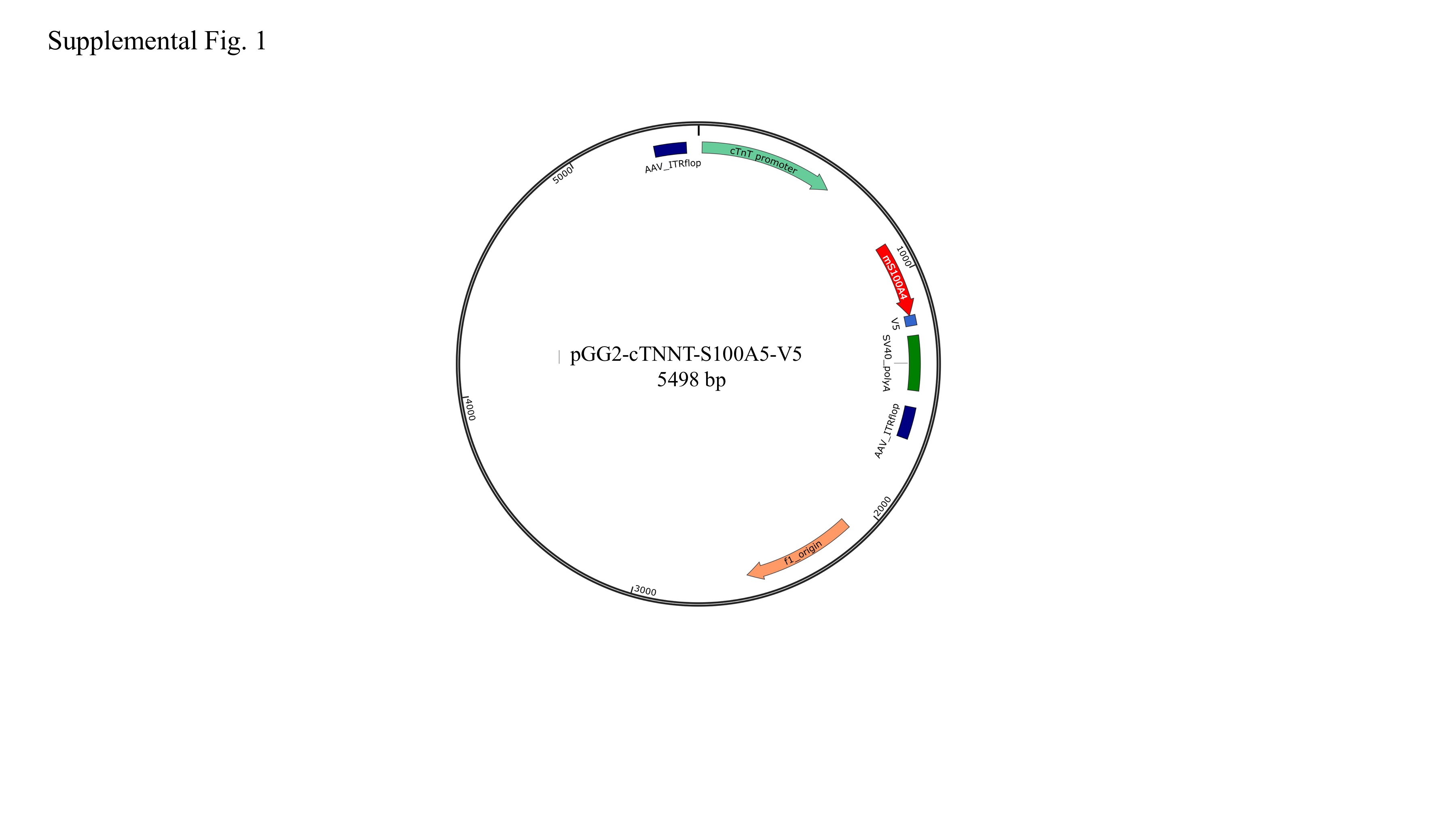

Supplement: Supplemental Figure 1 — Map of the plasmid used for the generation of AAV6, containing the C-terminally V5-tagged construct under the control of the human cardiac troponin T promoter (TNNT2). SV40_polyA sequence leads to polyadenylation to prevent rapid degradation, the two AAV_ITRflop sequences (inverted terminal repeats) are essential for AAV synthesis, the f1_origin allows for optional siRNA construction. [file Image_1.JPEG]

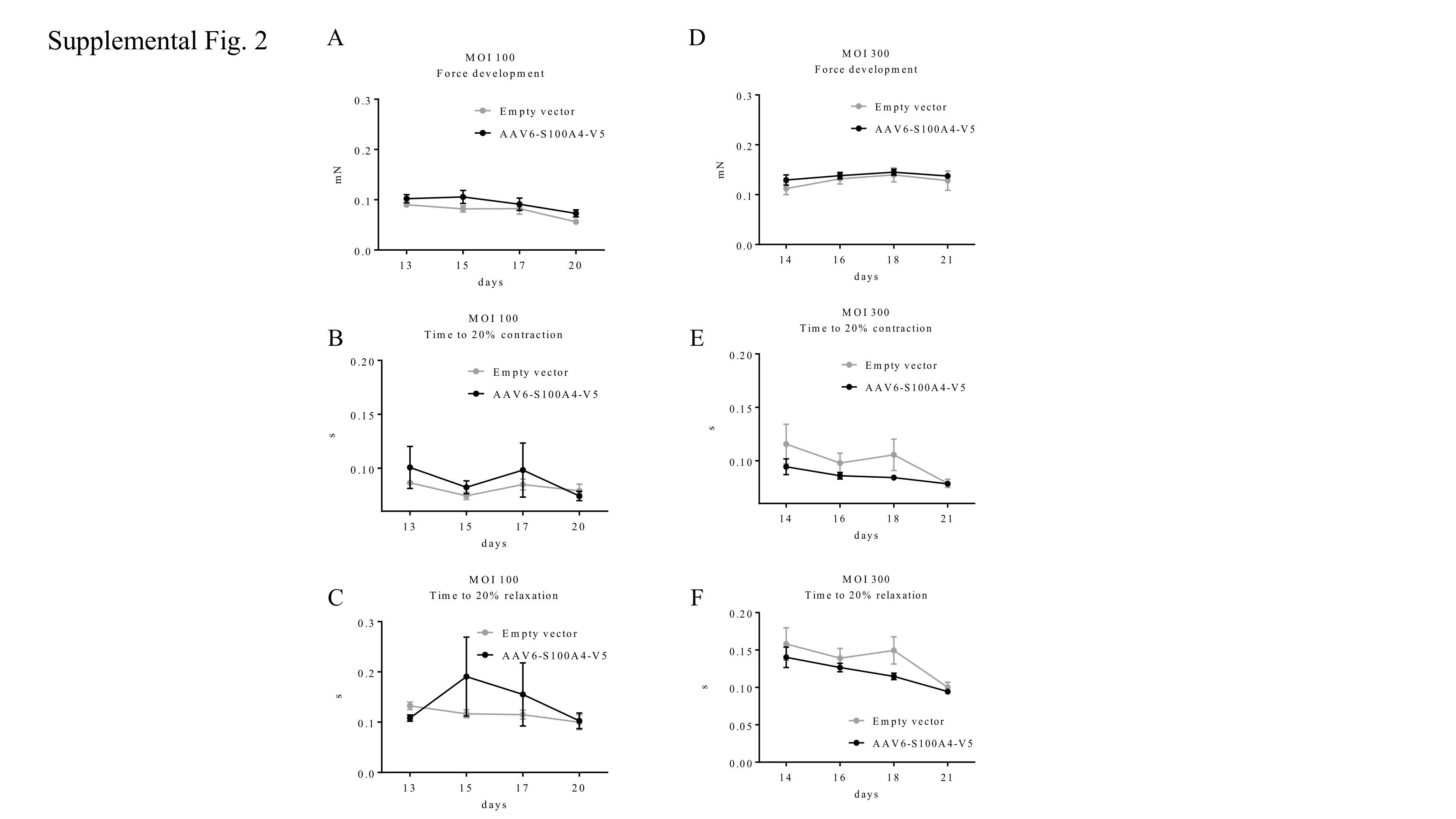

Supplement: Supplemental Figure 2 — Functional effects of AAV6-mediated overexpression of S100A4 (MOI 100, A–C and MOI 300, D–F) under the control of the human cardiac troponin T promoter (TNNT2) in engineered heart tissue (EHT) compared to EHTs treated with an empty vector to exclude side effects of the treatment. (A,D) Force development, (B,E) time to 20% contraction, i.e., TTP (−80%) or (C,F) relaxation, i.e., RT (80%). Data are expressed as mean ± SEM. One-way-ANOVA with Dunnett's-Multiple-Comparison-Test, n = 4–9 per group. [file Image_2.JPEG]

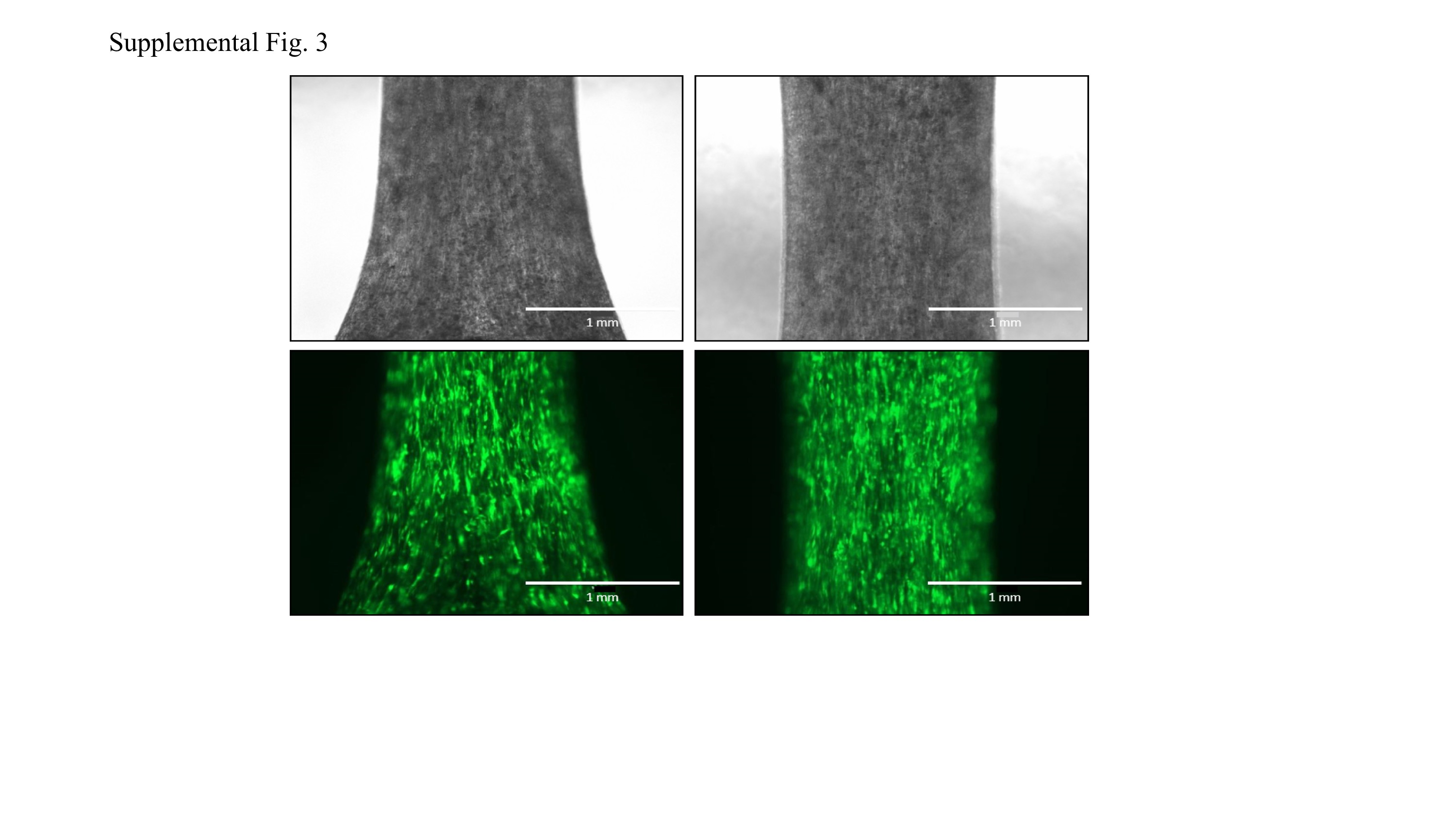

Supplement: Supplemental Figure 3 — Uncropped Western blots of Figure 1E. On the left staining for S100A4 (anti-S100A4 rabbit-polyclonal, Dako A5114, 1:500) on the right staining for ß-actin (anti-ß-actin mouse-monoclonal, 1:20,000). [file Image_3.jpg]

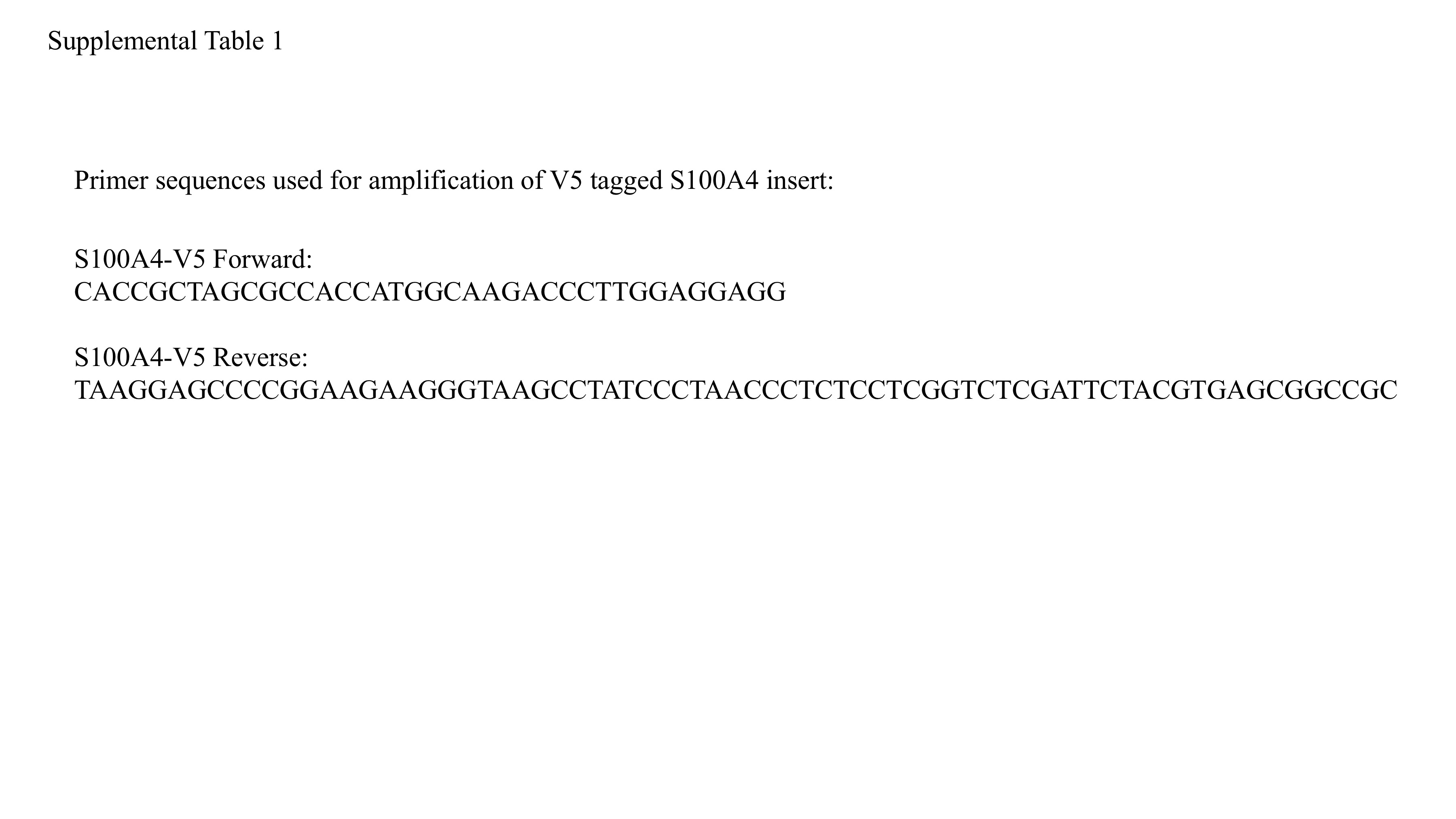

Supplement: Supplemental Table 1 — Sequences of primers chosen for amplification of V5-tagged S100A4 insert, later used for construction of respective plasmid and AAV6. [file Image_4.jpeg]
